# Changes in nutritional, health benefits, and pharmaceutical potential of raw and roasted tropical almond (*Terminalia catappa* Linn.) nuts from Nigeria

**DOI:** 10.1371/journal.pone.0287840

**Published:** 2024-01-02

**Authors:** Ifeoma Felicia Chukwuma, Emmanuel Chekwube Ossai, Florence Nkechi Nworah, Victor Onukwube Apeh, Emmanuel Osinachi Abiaziem, Franklyn Nonso Iheagwam, Hanna Skendrović, Szymon Juchniewicz, Katarzyna Leicht, Charles Odilichukwu R. Okpala, Małgorzata Korzeniowska

**Affiliations:** 1 Faculty of Biological Sciences, Department of Biochemistry, University of Nigeria, Nsukka, Enugu State, Nigeria; 2 Department of Applied Sciences, Federal College of Dental Technology and Therapy, Enugu State, Nigeria; 3 Department of Biochemistry, Covenant University, Ota, Ogun State, Nigeria; 4 Covenant University Public Health and Wellbeing Research Cluster (CUPHWERC), Covenant University, Ota, Ogun State, Nigeria; 5 Faculty of Food Technology and Biotechnology, University of Zagreb, Zagreb, Croatia; 6 Department of Functional Food Products Development, Wrocław University of Environmental and Life Sciences, Wrocław, Poland; 7 UGA Cooperative Extension, College of Agricultural and Environmental Sciences, University of Georgia, Athens, GA, United States of America; Bangabandhu Sheikh Mujibur Rahman Agricultural University, BANGLADESH

## Abstract

Tropical almond (*Terminalia catappa* Linn.) is highly distributed within the tropics, but appears rather underutilized in developing countries like Nigeria. Specifically, relevant information regards the nutritional, health benefits, and pharmaceutical potential of roasted *T*. *catappa* nuts remains scanty. Comparing both raw and roasted *T*. *catappa* nuts should provide additional information especially from product development and potential commercial prospect standpoints. The changes in nutritional, health benefits, and pharmaceutical potentials of raw and roasted *T*. *catappa* nuts were, therefore, investigated. Whereas the raw *T*. *catappa* nuts obtained significantly (p < 0.05) higher protein, ash, moisture, crude fiber, as well as vitamins C, and B_1-3_ compared to the roasted ones, some contents like carbohydrates, energy, vitamin A, calcium, manganese, zinc, hydrogen cyanide, as well as oxalate would noticeably change (p < 0.05) after the roasting process. Twenty phytochemicals were identified in both raw and roasted samples with the concentrations of quinine, ribalinidine, sapogenin, flavan-3-ol and tannin significantly reduced, while catechin seemed enhanced upon roasting. Promising drug-likeness, pharmacokinetic properties, and safety profiles could be predicted among the phytochemicals. Overall, roasting *T*. *catappa* nuts should enhance the nutritional contents, which could aid both absorption and palatability.

## Introduction

Identifying available and cheap nutrient-dense agro-foods could help solve the global burden of hunger/malnutrition, which have been triggered by the increasing population growth and unmatched food security plans. Developing healthy, quality, and safe food products from sustainable nutrient-dense agro-food sources is feasible via increased research, particularly those eco-friendly processes adaptable to consumers’ needs [1]. There is an increased global interest in nuts that remedies hunger and malnutrition given their enriched dietary fiber, high-quality plant protein, minerals, unsaturated fatty acids, vitamins, fat-soluble bioactives, and other phytonutrients [2–4]. Such phytoconstituents in nuts position them as promising agents that positively impact a myriad of health outcomes as well as natural pleiotropic nutraceuticals [3,5].

Nuts when incorporated as an essential constituent of human diet, either consumed whole (raw or roasted or salted) or as snack-type convenient food, contribute strongly to helping in sustaining healthy dietary patterns [4]. More so, the consumption of nuts would suppress hunger by modulating either the postprandial appetite or desire to eat, which creates a feeling of satiety [6]. More so, the processing methods applied to nuts generally enhances their organoleptic quality with long-term preservation [7]. From thermal (such as heating, blanching, roasting and frying) [8] to non-thermal (such as high hydrostatic pressure treatment and enzymatic treatment) types, the processing methods of nuts capably modify various characteristic qualities within the component matrix [7]. Given the aroma, flavor, texture, and color improvements that results, the roasting process continues to thrive as the increasingly preferred processing method for nuts [9].

Almond remains among the most consumed nut, especially in high-income countries. According to the International Nuts and Dried Fruits statistical yearbook 2018/2019, it accounted for about 39% of total consumption, with walnuts, cashews, and hazelnuts occupying the second, third, and fourth positions, respectively, in terms of consumption [10]. Tropical almond (*Terminalia catappa* Linn.) belongs to the *Combretaceae* family. Widely distributed within the tropics, including Nigeria, *T*. *catappa* plant thrives explicitly as shade at parks, along avenues as well as private gardens [11,12]. Besides its ellipsoid shape, the drupe of *T*. *catappa* plant is about 3–5.5 cm broad and 5–7 cm long, whereas its fruit, when ripe, turns from green to yellow or red [13]. Ripe mesocarp of *T*. *catappa* fruit is usually consumed by children. Further, the shell that is usually discarded covers the almond nut, the latter well established to comprise high protein, fat, carbohydrate, vitamin, and mineral contents [14–16].

However, relevant information regards the nutritional, health benefits, and pharmaceutical potential of roasted *T*. *catappa* nuts specific to Nigeria remains scanty. Essentially, the pharmacological and pharmacodynamic properties of nuts especially those possessing promising bioactive potentials need ascertaining through *in silico* integrative models of hits compounds. Achieving such feat (that is, the pharmacological and pharmacodynamic properties of nuts) especially within the biological system of drug candidates could help in predicting nut’s absorption, distribution, metabolism, excretion, and toxicity (ADMET) properties with high precision [17]. More so, to actualize compounds of potential bioavailability with less toxicity that could be re-purposed for drug development might help to cumulatively save cost as well as time spent in drug discovery that would fail at clinical trials. To supplement existing information, this current work investigated the changes in nutritional, health benefits, and pharmaceutical potential of raw and roasted *T*. *catappa* nuts from Nigeria. Importantly, comparing both raw and roasted *T*. *catappa* nuts should provide additional information especially from product development and potential commercial prospect standpoints.

## Materials and methods

### Schematic overview of the experimental design

**Fig 1:**
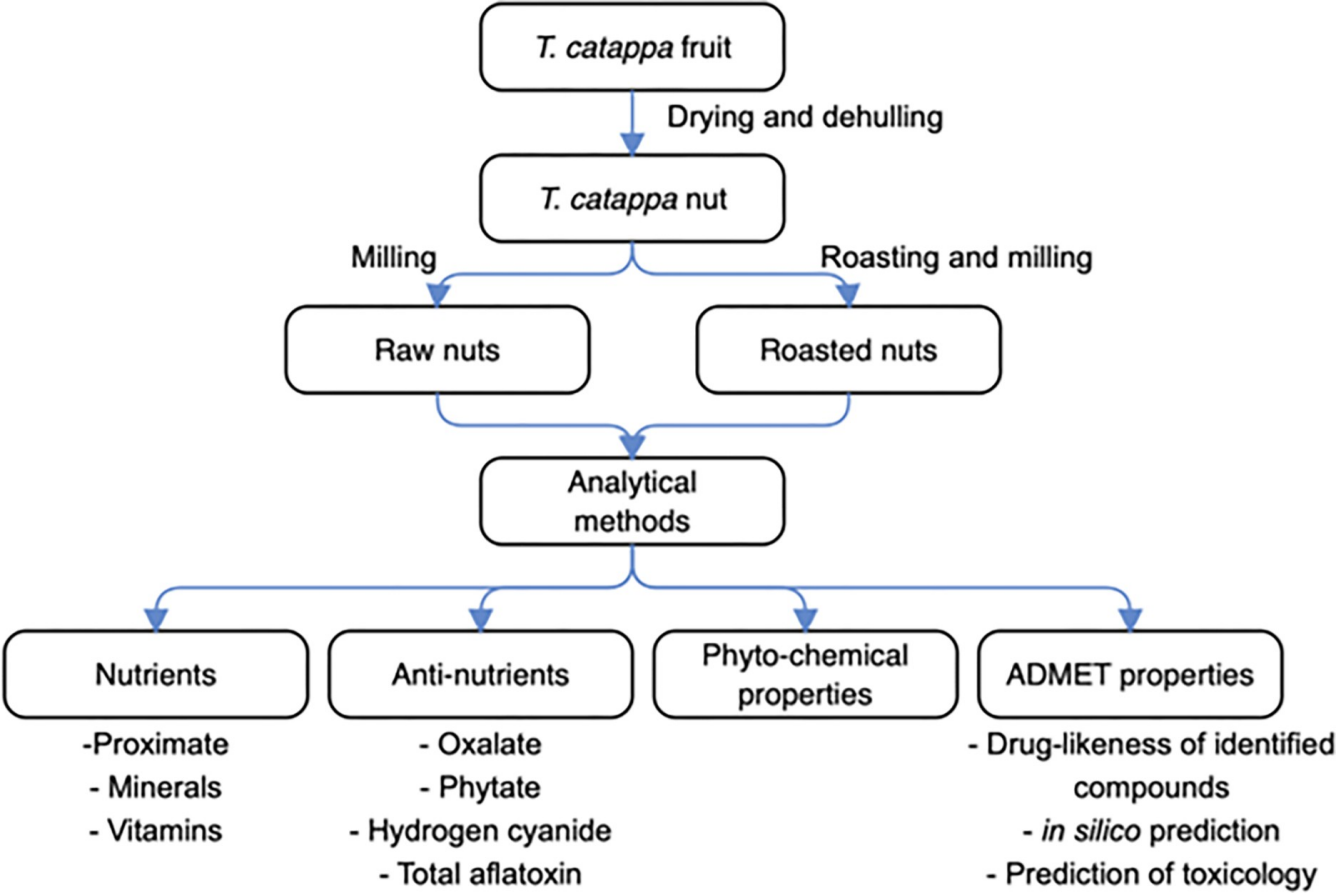
The schematic overview of the experimental design showing the key stages, from the drying and dehulling of *Terminalia catappa* fruits, roasting/milling of the nuts, analytical methods involving determinations of nutrients, anti-nutrients, phytochemical, and ADMET properties.

The schematic overview of the experimental design is depicted in **Fig 1**, which demonstrated the major stages, from the drying, and dehulling of *T*. *catappa* fruits, through the roasting/milling of nuts, to the analytical methods that involved the determinations of nutrients, anti-nutrients, phytochemical, and ADMET properties. For emphasis, this current work compared raw and roasted *T*. *catappa* nuts via nutritional (proximate, minerals, vitamins), anti-nutritional (oxalate, phytate, hydrogen cyanide, total aflatoxin), phytochemical, and ADMET (drug-likeness of the identified compounds, *in silico* prediction, and prediction of toxicity and toxicological effects) attributes. Importantly, all analytical measurements performed were in adherence to the relevant laboratory guidelines set out by the Department of Biochemistry, University of Nigeria, Nsukka, Enugu State, Nigeria.

### Chemicals and reagents

The chemicals and reagents used in this study were of an analytical grade standard. Products purchased from British Drug House, England include sulphuric acid, ammonium sulphate, aqua regia (a mixture of HNO_3_ and HCl in the ratio of 1:3), anhydrous sodium sulfate, concentrated tetraoxosulphate (VI) acid, nitric acid, silver trioxonitrate, trioxoborate (III) acid, diethyl ether; from May and Baker, England include iron (II) chloride, potassium ferric cyanide, potassium iodide, potassium tetraoxomanganate; from Merck, Germany include ammonium iron (III) sulphate, calcium (III) chloride, sodium hydroxide, ferric chloride, potassium chiocyanate; from Sigma-Aldrich, USA. include acetone, butylalcohol, heptane, hexane, ethanol, methanol, thioglycolic acid, ammonium thiocyanote, bipyridine, diphenol indo 2, 6 –dichlorophenol, potassium iodide, anhydrous sodium sulphate, isopropanol, 4-amino phenol, ammonium hydroxide, glacial acetic acid, hydrogen peroxide, oxalic acid, sodium acetate anhydrous, potassium hydroxide, a-a 1–dipyridyl, hydrocyanic acid, potassium sulfocyanate; from Abcam, USA, include the total aflatoxin ELISA kit (ab285282, type).

### Collection, preparation, and processing of *T*. *catappa* fruit samples

**Fig 2:**
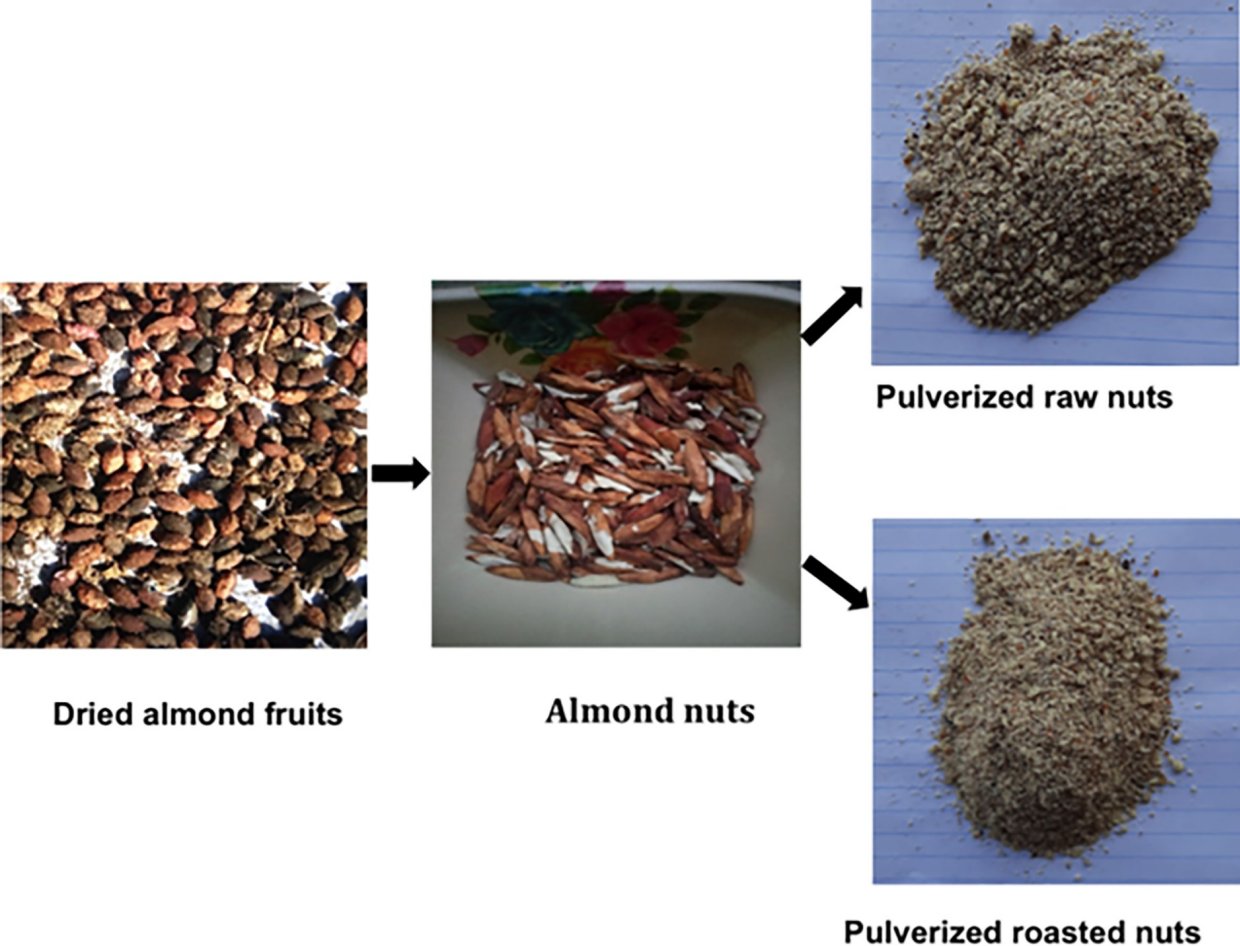
Pictorial display of almond fruits, almond nuts, pulverized raw and roasted almond nuts (*Terminalia catappa*).

For this research, the almond (*T*. *catappa*) fruits (red morphotypes) were harvested from the trees abundantly situated at Zuba, Gwagwalada Local Government Area, Federal Capital Territory (FCT)- Abuja, Nigeria (9.1023° N, 7.1952° E). Specifically, the *T*. *catappa* fruit harvest process adhered to institutionally prescribed plant material collection guidelines. Taxonomic identification of *T*. *catappa* fruit was conducted and a voucher specimen for reference purposes (voucher reference number PCG/UNN/0126) deposited in the herbarium of the Department of Pharmacognosy, Faculty of Pharmaceutical Sciences, University of Nigeria, Nsukka, Nigeria. Collected randomly from a batch, the almond fruits were first submitted to sun-drying for 14 days at temperature range of 34–40°C (93–104°F). The mesocarps were cracked open to remove the nuts and subsequently divided into two portions (500 g each) in line with the objective of this study. Specifically, one portion was designated for the raw sample and the other portion for the roasted sample. Following the method described by Tu et al. [9] with slight modifications, the nuts wrapped in aluminum foil were roasted using an electric rotary oven (Gallenkamp, England UK) at 125°C for 15 min based on the nut industry’s best practices. To prevent any quality deterioration, the roasting conditions applied were consistent with the good hygiene/manufacturing practices (GHP/GMP) prescribed by National Agency for Food and Drug Administration and Control (NAFDAC) for the Nigeria’s nut industry. The *T*. *catappa* nut samples were finely pulverized using a mechanical grinder (Vikas Ltd, England), and subsequently kept in a glass screw capped container at ambient temperature (25 ± 1°C) until required for further analyses. **Fig 2** shows the pictures of almond fruits, almond nuts, and pulverized raw and roasted almond nuts.

### Analytical measurements of raw and roasted T. *catappa* nuts

#### Determination of nutritional contents. Proximate components

The proximate components (crude protein, crude fats, ash, moisture, crude fiber, and carbohydrate contents) of samples were determined by the Association of Official Analytical Chemists (AOAC) method [18]. Crude protein content was determined using the Kjeldahl method that employed firstly, the determination of total nitrogen, and secondly, the conversion factor of 6.25. Crude fat content was determined using the Soxhlet extraction method by non-polar organic solvent, wherein the residue left after evaporation were calculated. Ash content was determined by direct analysis in a muffle furnace of 600°C to burn off all organic materials, leaving the inorganic materials that did not volatilize at this temperature (as designated ash). Moisture content was determined using the vacuum oven method and calculated as a weight change after oven drying (100°C). The acid and alkaline digestive methods determined fiber content; weight loss on the ignition after digestion of a moisture-free ether extract of the samples was taken as the crude fiber content. Finally, total carbohydrate content was obtained by subtraction the percentages of all the other food components (moisture + ash + fat + fiber + protein contents) from 100. All the proximate components were expressed as percentages (%). In contrast, the energy was calculated using individual caloric factors of carbohydrate (caloric factor = 4), protein (caloric factor = 4), and fats (caloric factor = 9) as shown in Eq (1) below.


Energy(kcal/100g)=(totalcarbohydrate×4)+(protein×4)+(fat×9) (1)

#### Mineral contents

The contents of selenium, calcium, copper, iron, manganese, and zinc in samples was determined according to AOAC [18] method with modifications using the atomic absorption spectrophotometer (AAS) model AA—7000 (Shimadzu, Japan). Pulverized samples (1 g each) were placed in a muffle furnace and heated for 3 h at 500°C. Subsequently, 5 mL of 1 N HNO_3_ was added to the cooled samples before evaporating them to dryness using a steam bath (Vikas Ltd, England). The dried samples were further heated for 10–15 min at 400°C until a greyish color was obtained. Then 20 mL of 1 N HCl was added to the cooled samples, and the resulting solution was filtrated into a 50 mL round volumetric flask. The obtained filtrate was used to perform analysis, followed concentration extrapolation from the standard curve automatically plotted by the instrument.

### Vitamin contents

To determine the vitamin A content, the AOAC [18] method with some modifications was used. A quantity of 1 g of each sample was weighed and added into a 100 mL flask fitted with a reflux condenser. Then, 20 mL of alcoholic sulphuric acid and absolute alcohol (10 mL) were added to the flask and wrapped with aluminum foil. The mixture was refluxed for 45 min and cooled. Following that, 5 mL of water was added into each flask and transferred to a separating funnel. Diethyl ether was used to extract non-saponified materials. The mixed ether extract was then washed to remove the acid and dried over anhydrous sodium sulphate. The extract was evaporated at low temperatures while protected from sunlight, with final traces of solvent removed in a stream of nitrogen and residues dissolved immediately in 10 mL isopropanol. The extinction of freshly prepared extract in isopropanol was measured at 325 nm against a solvent blank (T1) using a UV-spectrophotometer (Jenway 6305, Bibby Scientific Ltd, U.K.). Thereafter, samples were exposed to U.V. radiation until the extinction no longer decreased with time, and absorbance spectrophotometrically measured (T2). In the same way, the corresponding standard vitamin A solution was determined (for solvent blank = ST1; after exposure to radiation until extinction = ST2) using Eq 2.


$$Vitamin\;A(mg/100\;g)=\frac{T1‐T2}{ST1‐ST2}\times 1\times \mathrm{Dilution}\;\mathrm{factor}$$ (2)

To determine the vitamin B_1_ content, the AOAC [18] method with some modifications was used. Accurately, 2 g of each sample was weighed, then 0.5 mL of 4-amino phenol together with 5 mL of NH_4_OH (0.1 M), thereafter thoroughly mixed, followed by room temperature incubation for 5 min. Next, 10 mL of chloroform was added, which allowed for the observation of two layers. The absorbance of chloroform layer was measured at 430 nm against blank using a UV-spectrophotometer (Jenway 6305, Bibby Scientific Ltd, U.K.). Finally, the Vitamin B_1_ content was calculated using Eq 3 below.


$$Vitamin\;B_{1}(mg/100\;g)=\frac{\mathrm{Change}\;\mathrm{in}\;\mathrm{absorbance}\;\mathrm{of}\;\mathrm{sample}}{\mathrm{Change}\;\mathrm{in}\;\mathrm{absorbance}\;\mathrm{of}\;\mathrm{standard}}\times \mathrm{Concentration}\;\mathrm{of}\;\mathrm{standard}$$ (3)

To establish the vitamin B_2_ content, the AOAC [18] method was used with some modifications. The samples were weighed (2 g) and added to a calibrated test tube. In each test tube, 2 mL glacial acetic acid, 2 mL hydrochloric acid (1 M), 2 mL hydrogen peroxide, 2 mL potassium permanganate (15% w/v), and 2 mL phosphate buffer (pH 6.8) were added. The resulting mixture was mixed thoroughly before measuring its absorbance at 444 nm against blank using a UV-spectrophotometer (Jenway 6305, Bibby Scientific Ltd, U.K.). The content of vitamin B_2_ was calculated with the formula in Eq 4.


$$Vitamin\;B_{2}(mg/100\;g)=\frac{\mathrm{Change}\;\mathrm{in}\;\mathrm{absorbance}\;\mathrm{of}\;\mathrm{sample}}{\mathrm{Change}\;\mathrm{in}\;\mathrm{absorbance}\;\mathrm{of}\;\mathrm{standard}}\times \mathrm{Concentration}\;\mathrm{of}\;\mathrm{standard}$$ (4)

To determine the niacin content, the AOAC [18] method with some modifications was used. Samples (5 g) were autoclave digested with 1 N sulfuric acid with pH adjusted to 4.5 with 10 N sodium hydroxide, subsequently followed by filtration. Cyanogen bromide was complexed with the supernatants containing extracted niacin to form a purple color. Absorbance of solution was read at 470 nm against blank using a UV-spectrophotometer (Jenway 6305, Bibby Scientific Ltd, U.K.). The concentration of niacin was extrapolated from a standard curve.

To determine the vitamin C content, the AOAC [18] method with some modifications was employed. This involved the titration using diphenol indo 2, 6 –dichlorophenol (DPIP), which required sample (0.2 g) added to 4 mL of buffer solution (containing 1 g/L oxalic acid and 4 g/L sodium acetate anhydrous). The resultant solution was titrated against DPIP (295 mg/L) and sodium bicarbonate (100 mg/L). Vitamin C was calculated from Eq (5) below:

$$\mathrm{Vitamin}\;C(\mathrm{mg}/100\;g)=\frac{MV\times 100\times 100}{10\times B}$$ (5)

where: M = mass of ascorbic acid titrimetric equivalent to 0.001 M DPIP solution (mg)

100 is the dilution ratio of the sample taken. The second 100 is the scaling factor for conversion per 100 g of raw material, 10 is the titrate volume; V = titrant volume (0.00 1 M DPIP solution) ml; and B = weight of the sample extract used.

To determine the vitamin E content, the method described by Achikanu et al. [19] with some modifications was used. The sample (1 g) was macerated in 20 mL of n-hexane. Thereafter, the mixture was centrifuged for 10 min at 1500 rpm (4000 rpm, Abman, Canada), filtered, and treated with ethanol and alcoholic potassium hydroxide (0.5 N). Subsequently, 2 mL of the filtrate was evaporated to dryness in a boiling water bath (Gallenkamp, England). The residue was mixed with ethanol, ferric chloride (0.2%), and a-a 1-dipyridyl (0.5%). The absorbance of resultant solution was read at 520 nm against the blank using a UV-spectrophotometer (Jenway 6305, Bibby Scientific Ltd, U.K.), after which the concentration of vitamin E was calculated from Eq (6) below:

$$Vitamin\;E(mg/100\;g)=\frac{\mathrm{Absorbance}\;\mathrm{of}\;\mathrm{sample}\times \mathrm{Concentration}\;\mathrm{of}\;\mathrm{standard}}{\mathrm{Absorbance}\;\mathrm{of}\;\mathrm{standard}}$$ (6)

### Determination of anti-nutritional properties

*Oxalate content*. To determine the oxalate content, the titration method as described by Day and Underwood [20] with some modifications was used. A quantity of 1 g of the sample was added into a 100 mL conical flask. Then, 75 mL of 3 M H_2_SO_4_ was added to the flask, and stirring was done for 1 h with a hot plate with a magnetic stirrer, after which it was filtered with Whatman No 1 filter paper (pore size of 11 m). Immediately and still heated, 25 mL of the filtrate was titrated against 0.05 M KMnO_4_ resulting in a faint pink color chromophore that persists for 1 min. The oxalate content was then calculated by taking 1 mL of 0.05 M KMnO_4_ as equivalent to 2.2 mg oxalate using Eq 7.


$$Oxalate(mg/100\;g)=\frac{\mathrm{Titer}\;\mathrm{value}\times \mathrm{equivalent}\;\mathrm{weight}}{\mathrm{Weight}\;\mathrm{of}\;\mathrm{sample}\;\mathrm{used}}$$ (7)

*Phytate content*. To determine the phytate content, the method of Lucas and Markaka [21] with some modifications was used. To weighed 1 g sample, 100 mL of 2% HCl was added and allowed to stand at room temperature for 3 h before filtering through a double-layer filter paper. Thereafter, 25 mL of the filtrate was added. The solution’s acidity was improved with 53 mL of distilled water. A volume of 10 mL of 0.3% ammonium thiocyanate solution was added to each sample solution as an indicator before titrating with standard iron chloride solution (0.00195 g iron/mL), until endpoint (brownish-yellow coloration) persisted for 5 min. Phytate concentration expressed in mg/100 g was calculated with Eq 8.


$$Phytate(mg/100\;g)=\frac{\mathrm{An}}{\mathrm{As}}\times C\times \frac{100}{W}\times \frac{\mathrm{Vf}}{\mathrm{Va}}$$ (8)

Where A_n_ = absorbance of the test sample; A_s_ = Absorbance of the standard solution; C = concentration of standard solution; W = weight of the sample used; V_f_ = total volume of the extract; and V_a_ = volume of the extract analyzed.

*Hydrogen cyanide content*. To determine the hydrogen cyanide content, the AOAC [18] method with some modifications was used. This involved about 20 g of ground sample subject to distillation (2 h) to free all bound hydrocyanic acid, after which the collected distillate in 20 mL 0.01 N AgNO_3_ was acidified with 1 mL HNO_3_ (40 min). With 150 mL of distillate secured, filtration was performed with little water, followed by excess AgNO_3_ titrated with 0.02 N potassium sulfocyanate (KSCN) using a ferric alum indicator. The endpoint was observed (faint reddish color) upon the addition of 0.02 N KSCN. The volume of AgNO_3_ consumed by the complex was determined by the formula in Eq 9:

HCN=20−(2×V) (9)

where V = volume of titer; 1 mL of AgNO_3_ = 0.27 mg HCN per 100.

### Determination of total aflatoxin content

To determine the total aflatoxin content, the procedure described by Reza et al. [22] with some modifications, which involved enzyme-linked immunosorbent assay (ELISA) analysis and total aflatoxin ELISA kit (ab285282, Abcam, USA) The aflatoxin was extracted from 5 g of the samples using 33% methanol. The extract was filtered through Whatman no 1 filter paper. Then, 50 μL of the filtrate and 50 μL of 33% methanol, transferred to microtubes, were stored at -20°C until the analysis started. About 50 μL of distilled was added to the mixing well. Subsequently, 50 μL of varying standard concentrations namely, 0.1 μg/kg, 0.2 μg/kg, 0.5 μg/kg, and 1.4 μg/kg, were added to samples wells. By pipetting 50 μL of enzyme conjugate and 100 μL antibody (anti-aflatoxin) into all the wells, the reaction was initiated, gently mixed, and incubated at room temperature for 20 min. The content of the antibody–coated well was emptied, and washed intermittently, Afterwards, 200 μL of chromogen solution was added to each well and incubated at room temperature (20 min), then adding 50 μL of stop solution, followed again by gently mixing. The absorbance of aflatoxin in the samples and standard were read with an ELISA microplate reader (State Fax^®^ 2100, Awareness, USA) at 450 nm. Thereafter, the total aflatoxin contents of each sample were calculated from a standard curve.

### Characterization of phytochemical composition by GC-FID

The characterization of phytochemical compounds followed the method described by Bezerra and Filho [23] with some modifications. Specifically, the Shimadzu G.C. 2010 gas chromatograph directly connected to a mass spectrometer (GCMS), has been equipped with a flame ionization detector (FID). In addition to the injection mode, the carrier gas used helium. The mass spectra were kept in electron impact mode between 40 and 600 amu using a quadruple analyzer. In the end, the phytochemical contents in raw and roasted samples were reported in μg/mg based on the peak area produced in the chromatogram.

#### Determination of ADMET properties

*Drug-likeness of the identified compounds*. The canonical smiles of the identified compounds retrieved from the PubChem database (www.pubchem.com) were used to predict the compound’s absorption, distribution, metabolism, and excretion (ADME) using the SwissADME web server (http://www.swissadme.ch/). The physicochemical properties of molecular weight (M.W.), no of rotatable bond (Nrot), Hydrogen Bond Donor and Acceptor (HBD and H.B.A.), Topological Polar Surface Area (TPSA), n-octanol/water partition coefficient (Log P) were subjected to drug-likeness filters proposed by Egan et al. [24], Veber et al. [25], and Lipinski et al. [26].

*In silico prediction of the pharmacokinetics of the identified compounds*. Oral bioavailability and brain barrier permeability were predicted with lipophilicity profiles (WLogP) and polarity (TPSA) using the method of Brain or Intestinal Estimation Permeation (BOILED-Egg) from the Swiss ADME platform. At the same time, P-glycoprotein inhibition/substrate, as well as cytochrome P450 inhibition and skin permeation, were also estimated from the SwissADME platform (http://www.swissadme.ch/) [27].

*Prediction of toxicity and toxicological effects of the identified compounds*. ProTox-II webserver (https://tox-new.charite.de/protox_II/) was employed to predict toxicity and toxicological effects of the identified compounds, which is considered an essential part of the drug design development process. The ProTox-II web server incorporates pharmacophores, molecular similarity, fragment propensities, and machine-learning models to predict oral toxicity, organ toxicity (hepatotoxicity), and toxicity endpoints (carcinogenicity, immunogenicity, mutagenicity, and cytogenecity).

### Statistical analysis

All data of triplicate determination were subject to Student T-test. The emergent results were presented as mean±standard deviation (SD). The level of statistical significance was set at p < 0.05 (95% confidence level). Statistical Package for the Social Sciences (SPSS) for Windows version 23 (IBM® SPSS Inc., Chicago, IL- USA.) was used to run the data.

## Results and discussion

### Changes in nutritional value

Nutritional value of raw and roasted *T*. *catappa* nut samples, including proximate composition and energy, vitamins, and minerals were depicted in **Tables 1–3**. Significant differences (p < 0.05) across proximate components happened comparing raw and roasted nuts, with the exception of crude fat (p > 0.05). Specifically, raw *T*. *catappa* nuts’ crude protein (31.24 ± 0.75%), ash (7.25 ± 0.14%), moisture (10.01 ± 0.32%) and crude fiber contents (5.00 ± 0.24%) were significantly higher (p < 0.05) than those of roasted (crude protein content = 27.56 ± 1.96%; ash content = 6.76 ± 0.23%; moisture content = 5.38 ± 0.04%; and crude fiber content = 2.23 ± 0.06%). High ash content depicts the total amount of minerals present [28] in roasted *T*. *catappa* nuts, which appeared comparable with those of macadamia nuts [9]. Moreover, the progress of Maillard reaction may relate with decreasing crude protein herein, potentially rendering the amino acid inaccessible for digestion [15]. High protein content of *T*. *catappa* nut might help avert the global challenge of protein malnutrition [29]. By facilitating both cholesterol excretion and food digestion, crude fiber enhance the immune system [28]. Favorably, crude protein of raw and roasted *T*. *catappa* compared well with other conventional oilseeds like black-eyed beans (27.13%), brown beans (28.00%), cowpea (27.80%), groundnut (25.00%) Bambara nuts (23.41%), and pigeon pea (21.88%) [13]. Roasted *T*. *catappa* nuts obtained significantly higher (p < 0.05) carbohydrate (38.68%) and energy (439.2 kcal/100 g) compared to raw (carbohydrate content = 25.10%; energy content = 417.6 kcal/100 g) samples. These (carbohydrate and energy) results suggest thermal processing method of roasting able to modify the biochemical characteristics within the nut matrix [7,8]. Despite the somewhat high energy content of raw and roasted samples [14], the proximate data herein corroborated those found in sesame varieties [30], but contrary to those in sunflower seeds [31].

**Table 1:** Changes in proximate composition and energy of raw and roasted *Terminalia catappa* nut samples.

Proximate composition	Raw nut	Roasted nut	p -values
Protein (%)	31.24 ± 0.75^b^	27.56 ± 1.96^a^	0.039
Crude fat (%)	21.38 ± 0.90^a^	19.52 ± 1.60^a^	0.155
Ash (%)	7.25 ± 0.14^b^	6.76 ± 0.23^a^	0.036
Moisture (%)	10.01 ± 0.32^b^	5.38 ± 0.04^a^	0.000
Crude fiber (%)	5.00 ± 0.24^b^	2.23 ± 0.06^a^	0.001
Carbohydrate (%)	25.10 ± 1.10^a^	38.68 ± 0.61^b^	0.000
Energy (kcal/100 g)	417.6 ± 2.50^a^	439.2 ± 8.10^b^	0.012

Data represent mean ± standard deviation (SD) of triple measurements. Values with different superscripts in a row are statistically different at p < 0.05.

**Table 2:** Changes in vitamin components of raw and roasted *Terminalia catappa* nut samples.

Vitamin contents (mg/100 g)	*Raw nuts	*Roasted nuts	p-values	^#^RNI (Adult, 19^+^ years) References Male Female
A (Retinol)	0.23± 0.20^a^	0.28± 0.01^b^	0.018	0.9 0.7mg/day (Agbai et al., 2021)
E (Carotenoid)	24.77± 2.25^a^	26.00± 1.00^a^	0.437	10 8 mg/day (RDA, 1989)
B_1_ (Thiamin)	0.32± 0.03^b^	0.15± 0.04^a^	0.004	1.2 1.1 mg/day (FAO/WHO, 2001)
B_2_ (Riboflavin)	1.14± 0.02^b^	0.67± 0.10^a^	0.001	1.3 1.1 mg/day (FAO/WHO, 2001)
B_3_ (Niacin)	3.52± 0.10^b^	2.52± 0.20^a^	0.001	16 14 NEs (FAO/WHO, 2001)
C (Ascorbic acid)	0.10± 0.01^b^	N.R.	0.000	45 45 mg/day (FAO/WHO, 2001)

Key

*Data of the current study

^**#**^ Published references; N.R.: No record; RNI: Recommended nutrient intake; NEs: Niacin equivalent; Data represent mean ± standard (SD) of triple measurements. Values with different superscripts in a row are statistically different at p < 0.05.

**Table 3:** Changes in mineral composition of raw and roasted *Terminalia catappa* nut samples.

Minerals (ppm)	*Raw nuts	*Roasted nuts	p -values	^#^RNI (Adult, 19^+^) References Male Female
Selenium	0.45 ± 0.03^a^	0.42 ± 0.03^a^	0.288	34 26 μg/day (RDA, 1989)
Calcium	56.67 ± 1.00^a^	77.32 ± 1.80^b^	0.000	1000 1000 mg/day (RDA, 1989)
Copper	0.03 ± 0.01^a^	ND	0.000	900 900 μg/day (RDA, 1989)
Iron	1.22 ± 0.04^a^	1.22 ± 0.60^a^	1.000	10 15 mg/day (RDA, 1989)
Manganese	0.03 ± 0.01^a^	0.16 ± 0.01^b^	0.000	5.0 2.0 mg/day (RDA, 1989)
Zinc	0.26 ± 0.03^a^	0.32 ± 0.01^b^	0.027	15 12 mg/day (RDA, 1989)

**Key**

*Data of the current study

^**#**^ Published references; ND = Not detected; RNI = Recommended nutrient intake; Data represent mean ± SD of triple determination; Values with different superscripts in a row are statistically different at p < 0.05.

As shown in **Table 2**, roasted *T*. *catappa* nuts obtained significant increases (p < 0.05) in vitamins A (0.28 ± 0.01 mg/100 g) and E (26.00 ± 1.00 mg/100 g) and at the same time, reductions (p < 0.05) in B_1-3_ (vitamin B_1_ = 0.15 ± 0.04 mg/100 g; vitamin B_2_ = 0.67 ± 0.10 mg/100 g; vitamin B_3_ = 2.52 ± 0.20 mg/100 g) compared to the corresponding values in raw samples (vitamin A = 0.23 ± 0.20 mg/100 g; vitamin E = 24.77 ± 2.25 mg/100 g; vitamin B_1_ = 0.32 ± 0.03 mg/100 g; vitamin B_2_ = 1.14 ± 0.02 mg/100 g; vitamin B_3_ = 3.52 ± 0.10 mg/100 g). High temperatures emanating from roasting probably decreased the water-soluble vitamin concentrations [15]. Typically, vitamin A would enhance good vision, cell growth, and provide a healthy immune system. Herein, vitamin E of roasted *T*. *catappa* nuts compared favorably with previously published data of walnuts (0.70 mg/100 g), cashew (0.90 mg/100 g), pistachio (2.86 mg/100 g), pecans (1.40 mg/100 g), pine nuts (9.33 mg/100 g) and hazelnuts (15.03 mg/100 g) [32]. Moreover, thiamin, riboflavin, and niacin of roasted *T*. *catappa* nuts were significantly (p < 0.05) lower than the raw samples, probably owed to their vulnerability to high temperatures. Additionally, to extend the roasting duration should further destabilize the chemical bonds of methylene group [33]. Whilst vitamin B group acts as co-enzymes in various metabolic pathways [1], the vitamin C scavenges reactive species to maintain redox equilibrium [34], and therefore, their presence in *T*. *catappa* nuts would help especially in managing metabolic aberrations.

As shown in **Table 3,** different minerals were detected, which included selenium (Se), calcium (Ca), copper (Cu), iron (Fe), manganese (Mn), and zinc (Zn). Specifically, only Ca, Mn and Zn composition of raw samples differed noticeably (p < 0.05) from the roasted ones. The obtained minerals compared favorably with the recommended nutrient intake (RNI) values [35]. As co-factor for antioxidant enzymes, Se would protect the human body from oxidative damage [36]. Being somewhat high in the roasted nuts, the Ca alongside lower oxalate, not only confirms an inverse relationship to bring about calcium oxalate, but further enhances the function of membrane permeability, as well as the absorption of Zn and Mg [37]. Vital in the formation of myelin sheaths of nervous system, Cu helps the absorption of Fe into the gastrointestinal tract (GIT) [38]. Herein, the Fe concentration (1.22 ppm) suggests *T*. *catappa* nuts with promising anti-anemic potential for red blood cell formation [39], alongside immune function, and cognitive development [40]. Whilst Mn promotes the biosynthesis of proteoglycan in cartilage and useful in pyruvate metabolism and urea formation [38], the Zn aids the biochemical pathways involved in immunity, reproduction, and sexual development [40]. Overall, the above-mentioned minerals help to maintain the biochemical activities, especially those involved in enzyme function as well as tissue homeostasis [36].

### Changes in anti-nutritional and total aflatoxin values

As shown in **Table 4**, raw and roasted *T*. *catappa* nut samples contained quantifiable amounts of phytates, oxalate, hydrogen cyanide (HCN), and total aflatoxins, all of which were within the prescribed safety limits [1,41–43]. Specifically, whilst phytates of raw resembled (p > 0.05) those of roasted samples, the latter obtained significantly higher (p < 0.05) oxalate, HCN and total aflatoxins. Above-mentioned post-roasting differences in anti-nutrient (oxalate, HCN and total aflatoxins) might explain their thermolabile nature [15,41], hence, improving both nutrient bioavailability/digestibility and consumer safety of *T*. *catappa* nuts. Besides, phytate and oxalate values reported in *Passiflora edulis* var. *flavicarpa* (0.47 ± 0.02 mg/100 g) [44] fell below those in *T*. *catappa* nuts. Besides, high concentration of Ca^2+^ facilitating the decreasing phytic acid [15] in roasted *T*. *catappa* nuts, the reduced HCN demonstrates the usefulness of high temperatures to progress cellular inactivation of cytochrome oxidase within the mitochondria [44].

**Table 4:** Changes in anti-nutrient and total aflatoxin contents of raw and roasted *Terminalia catappa* nut samples.

Contents	Raw nuts *	Roasted nuts*	p -values	Safety limits^#^	References of the safety limits ^#^
Phytate (mg/100 g)	0.10 ± 0.02^a^	0.06 ± 0.02^a^	0.070	< 25	(Coulibaly et al., 2011)
Oxalate (mg/100 g)	0.08 ± 0.01^b^	0.03 ± 0.02^a^	0.018	< 10	(Agbai et al., 2021)
Hydrogen cyanide (mg/100 g)	0.20 ± 0.50^b^	0.10 ± 0.40^a^	0.000	1	(FAO/WHO, 2011)
Total aflatoxin (μg/kg)	5.66 ± 0.35^b^	2.77 ± 0.26^a^	0.000	**<** 8	(EFSA, 2007)

Key

*Data of the current study

^**#**^ Published data and references; Data represent mean ± SD of triple determination. Values with different superscripts in a row are statistically different at p < 0.05.

The toxic nature of anti-nutritional factors negate both availability of minerals, and digestibility of proteins [45,46]. As a deprotonated (salt) form of dodecaprotic phytic acid, the phytates capably reduce the absorption/bioavailability of proteins, carbohydrates, lipids, and divalent minerals via electrostatic interactions that involve coordination complexes [45,46]. High temperatures to significantly reduce moisture, and denature the protein contents in roasted *T*. *catappa* nut further substantiate the elimination and detoxification of such anti-nutritional contents like HCN [46]. Moreover, the HCN values of *T*. *catappa* nut (shown in **Table 4**) appear comparable with those of raw walnut (0.112 ± 0.10 mg/100 g) [39]. Likewise, the total aflatoxin contents in *T*. *catappa* nut (raw = 5.66 ± 0.35 μg/kg; roasted = 2.77 ± 0.26 μg/kg) were below the safety limits (8 μg/kg) established by the European Commission for total aflatoxins in ready-to-eat almonds, hazelnuts, and pistachios [42]. Elsewhere, thermal processing specifically roasting has been shown to reduce the anti-nutritional content to permissible levels [46], which further buttresses the suitability of *T*. *catappa* nut for consumption (**Table 4**). According to Joint FAO/WHO Expert Committee on Food Additives (JECFA), aflatoxins are among well-known mutagenic substances and group 1 carcinogens [22].

### Phytochemical characterisation by GC-FID

**Fig 3:**
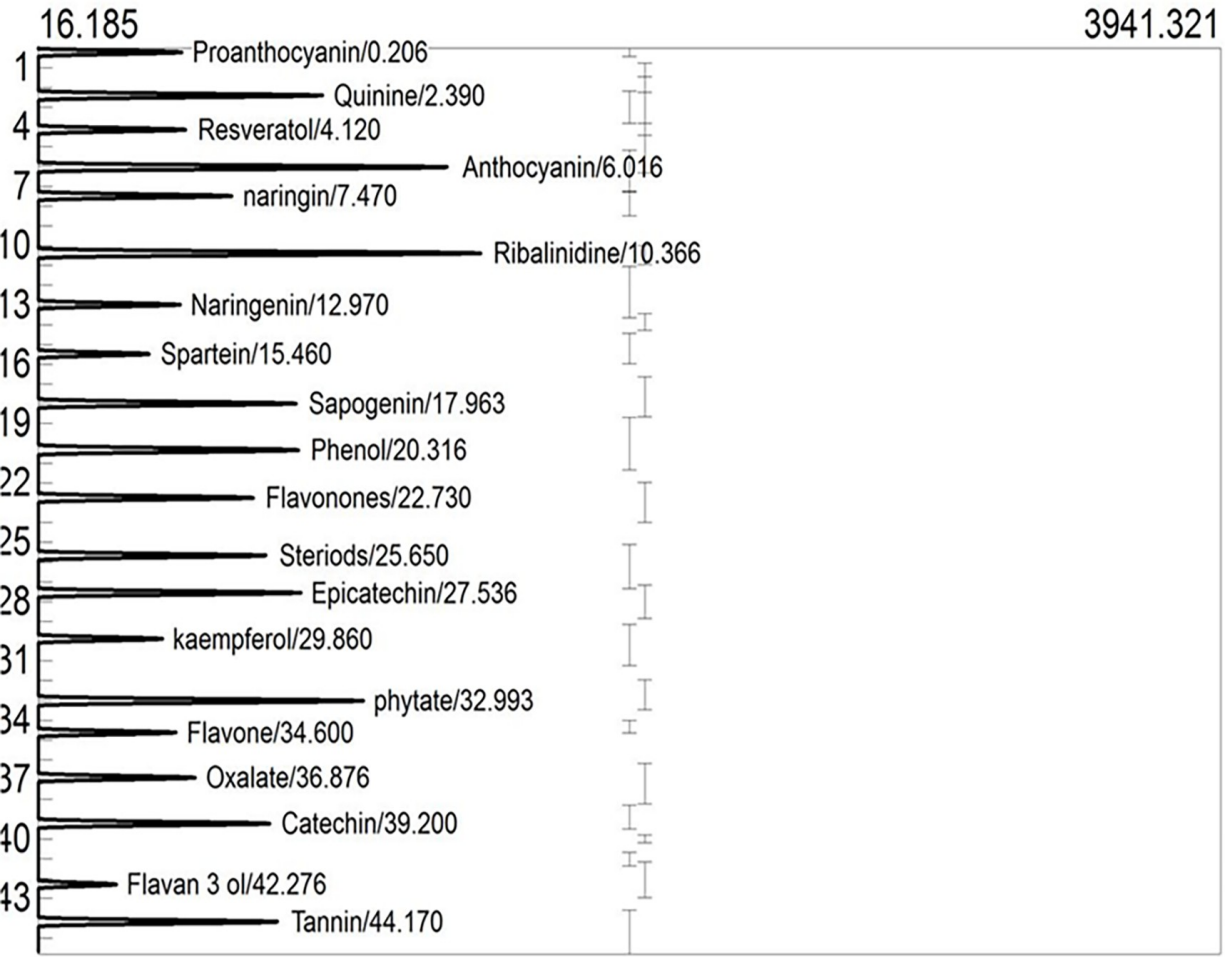
GCMS of phytochemicals in raw *Terminalia catappa* nuts.

**Fig 4:**
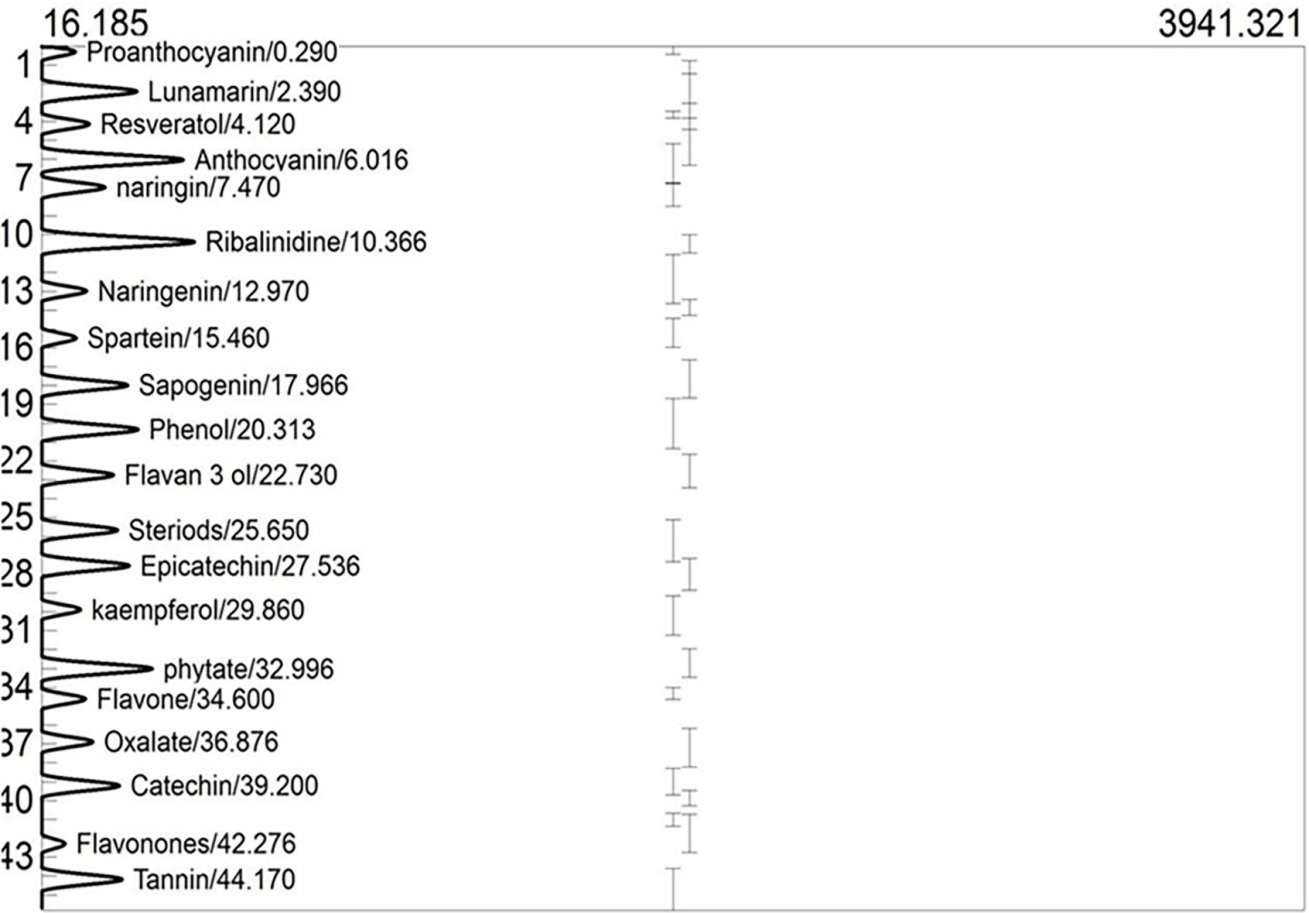
GCMS of phytochemicals in roasted *Terminalia catappa* nuts.

GCMS profiles of raw and roasted nuts are respectively shown in **Figs 3 and 4**. Phytochemical characteristics of raw and roasted nuts are shown in **Table 5.** The *T*. *catappa* nuts reveal a promising abundance of flavonoids (proanthocyanin, naringin, flavan-3-ol, anthocyanin, naringenin, flavanones, epicatechin, kaempferol, flavone, catechin), phenolics (phenol), stilbenes (resveratrol), polyphenol (tannins), alkaloids (ribalinidine, quinine and sparteine), and saponin (sapogenin) together with phytate, and oxalate. Naringin and its aglycone form naringenin have been explored for their anti-inflammatory, antioxidant, nephroprotective, immunomodulatory, hepatoprotective, antidiabetic, anticancer, and anti-atherosclerotic attributes [47–51]. In addition to being an effective anti-malaria drug, quinine helps in treating diarrhea, whereas ribalinidine, a quinoline alkaloid, possesses radical scavenging capacity [38]. In addition to flavan-3-ol monomers and oligomers with antioxidant potential [52], flavanones possess antioxidant, antihyperlipidemic, and anti-inflammatory effects, whereas flavones have antimicrobial and antifungal properties [38].

**Table 5:** Phytochemical characteristics of raw and roasted *Terminalia catappa* nut samples.

Peaks	Identified Compounds	Molecular Formulae	Molecular Weights (g/mol)	Conc. (μg/g)		p-values	Class of compounds
				Raw	Roasted		
1	Proanthocyanin	C_31_H_28_O_12_	594.50	10.46^a^	8.13^a^	0.147	Flavonoid
2	Naringin	C_27_H_32_O_14_	580.54	12.71^a^	12.57^a^	0.919	Flavonoid
3	Quinine	C_20_H_24_N_2_O_2_	324.42	7.18 ^b^	0.46^a^	0.000	Alkaloid
4	Flavan-3-ol	C_15_H_14_O_2_	456.39	32.67 ^b^	24.41^a^	0.007	Flavonoid
5	Anthocyanin	C_15_H_11_O	207.30	7.14^a^	7.11^a^	0.972	Flavonoid
6	Ribalinidine	C_15_H_17_NO_4_	275.30	45.57 ^b^	17.07^a^	0.000	Alkaloid
7	Naringenin	C_15_H_12_O_5_	272.26	8.04^a^	8.02^a^	0.988	Flavonoid
8	Spartein	C_15_H_26_N_2_	234.38	7.33^a^	7.31^a^	0.988	Alkaloid
9	Sapogenin	C_58_H_94_O_27_	490.70	22.76 ^b^	13.45^a^	0.007	Saponin
10	Phenol	C_6_H_6_O	94.11	15.66^a^	15.65^a^	0.846	Phenolics
11	Flavonones	C_29_O_11_H_27_	594.50	9.97^a^	9.96^a^	0.991	Flavonoid
12	Steroid	C_19_H_28_O_2_	288.40	17.35^a^	17.24^a^	0.899	Steroids
13	Epicatechin	C_15_H_14_O_6_	290.26	18.08^a^	17.99^a^	0.959	Flavonoid
14	Kaempferol	C_15_H_10_O_6_	286.23	7.08^a^	7.07 ^a^	0.994	Flavonoid
15	Phytate	C_6_H_18_O_24_P_6_	660.04	9.68^a^	9.50 ^a^	0.918	Anti-nutrient
16	Flavone	C_15_H_10_O_2_	222.24	5.44 ^a^	3.48 ^a^	0.074	Flavonoid
17	Oxalate	C_2_O_4(2-)_	128.09	13.67^a^	13.65^a^	0.988	Anti-nutrient
18	Catechin	C_15_H_14_O_6_	290.26	6.02^a^	14.44^b^	0.000	Flavonoid
19	Resveratrol	C_14_H_12_O_3_	228.25	6.02^a^	5.67^a^	0.954	Stilbenes
20	Tannin	C_76_H_52_O_46_	1701.19	27.13 ^b^	22.54^a^	0.024	Polyphenol

Values with different superscripts in a row are statistically different at p < 0.05.

**Fig 5:**
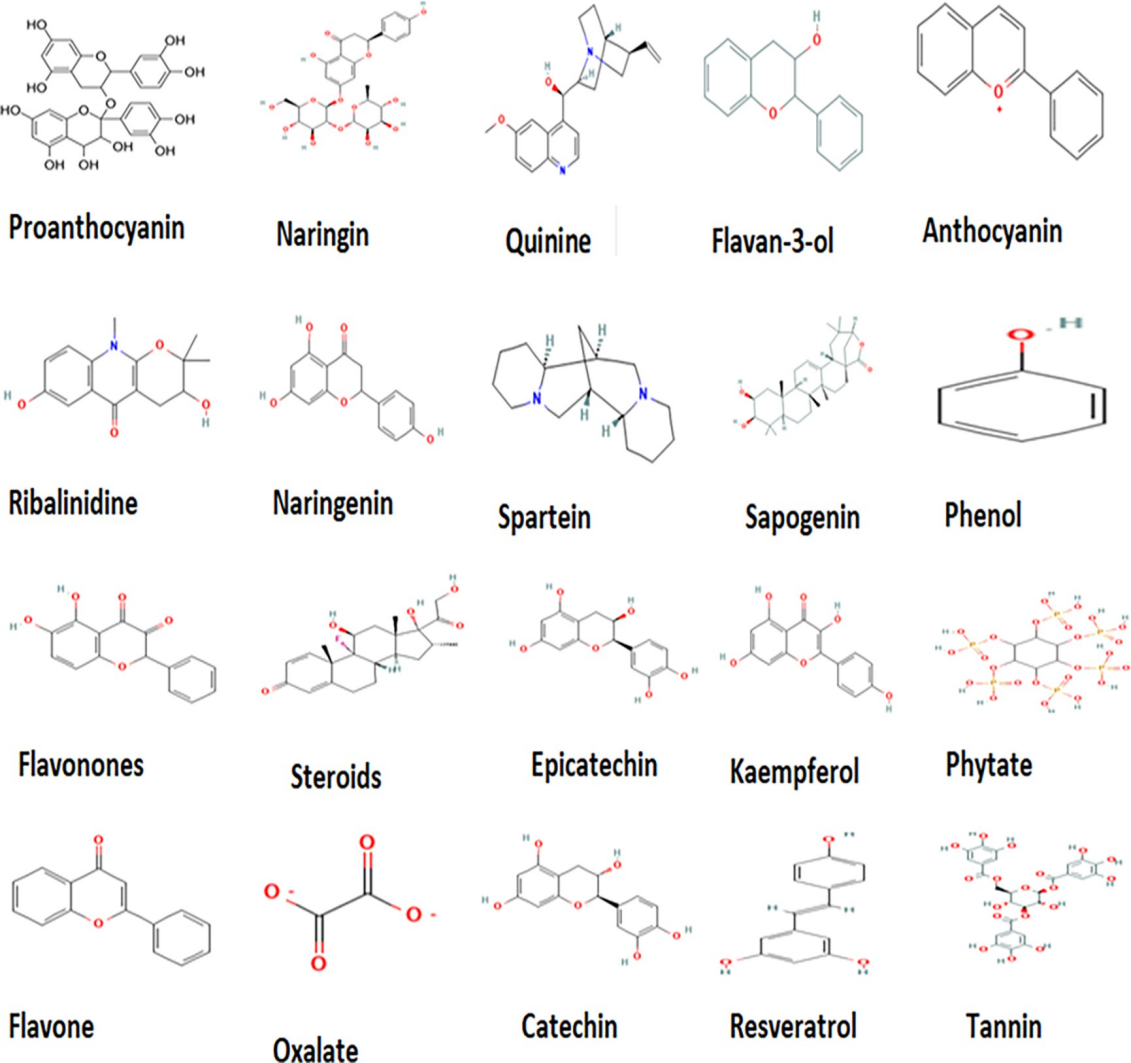
2D structures of identified compounds from *Terminalia catappa* as retrieved from PubChem database.

The 2D structures of identified compounds from *T*. *catappa* nuts as retrieved from the PubChem database is shown in **Fig 5**. The presence of hydroxyl, carbonyl, phenyl, amino, and carboxylic acids functional groups might be strengthening the therapeutic efficacy of these phytocompounds, which by acting as reducing agents, would stabilize the redox equilibrium—a precursor for averting the onset of metabolic diseases [28]. Epicatechins possess antimicrobial, antioxidant, cardioprotective, antidiabetic, and anticancer properties [53]. Whilst *in vivo* platelet anti-aggregation and improved insulin sensitivity have been associated with epicatechin-rich green tea [54], catechins demonstrate anti-obesity, anticancer, hepatoprotective, antidiabetic, antioxidant, and neuroprotective properties [55]. Whereas anthocyanin is an effective agent against the onset of cardiovascular diseases [56], the kaempferol is an aglycone flavonoid with anticancer, antioxidant, anti-inflammatory, and antidiabetic promise [34]. Resveratrol—a stilbene polyphenol, would not only prevent cerebrovascular/cardiovascular diseases, but also resist oxidation, and lower inflammation, obesity, diabetes, and fibrosis [57,58]. The bioactive phytochemicals present in almond nuts, as above-mentioned, should enhance human health after consumption at safety limits. From **Table 5** and upon roasting, the concentration of quinine (0.46 μg/g), ribalinidine (17.07 μg/g), sapogenin (13.45 μg/g), flavan-3-ol (24.41 μg/g) and tannin (0.46 μg/g) significantly reduced, whereas catechin (14.44 μg/g) significantly increased. Whilst both quinine and ribalinidine are considered as alkaloids, both sapogenin and flavan-3-ol are deemed respectively as saponins and flavonoids, able to confer a strong bitter taste despite their health-promoting benefits [59,60]. When tannins appear in large quantities, a high astringency/unpleasant flavor would emerge compared to lower amounts easily tolerated [61]. The fact that roasting process of this current study was able to alter the phytoconstituents could depict biochemical change within the food matrix that enhance both flavor and palatability of nut for consumers.

### Changes in ADMET properties

ADMET prediction represents a fast yet low-cost approach to evaluate the potential of easily absorbable drug, which should be well-distributed to its target action site, favorably metabolized, and easily eliminated from the body without toxic side effects [62,63]. The predicted drug-likeness of the compounds identified from *T*. *catappa* nuts is shown in **Table 6**. Interestingly but except proanthocyanin, naringin, phytate and tannin, most others appear as available promising oral drugs that do not violate Lipinski, Veber, and Egan drug-likeness parameters having favorable bioavailability score of 0.55. Low polarity of these compounds (TPSA ≤ 140°A) equally portrays a higher interaction with therapeutic targets involved in several diseases [34]. Bioavailability of drug candidates should better predict new drug discovery via the Lipinski “rule of five” (MW ≤ 500; Log P ≤ 5; HBA ≤ 10; HBD ≤ 5) [26], in addition to Nrot ≤ 10 and TPSA ≤ 140°A° as depicted by the rules of Egan et al. [24] as well as Veber et al. [25]. It appears pretty clear that these (above-mentioned) compounds appear not violating these cut-off values, hence, are suggested as excellent oral drug candidates [34]. High rate of clinical failures for orally available drugs are associated with poor availability/efficacy when administered to humans [64].

**Table 6:** Predicted drug-likeness of the compounds identified from raw and roasted *Terminalia catappa* nuts.

Compounds	MW (g/mol)	Log P	#HBA	# HBD	Nrot	TPSA	BAS	# Violations		
								Lipinski	Veber	Egan
Proanthocyanin	593	2.29	12	9	4	209.8	0.17	3	1	1
Naringin	581	2.38	14	8	6	225	0.17	3	1	1
Quinine	324	3.36	4	1	4	45.59	0.55	0	0	0
Flavan-3-ol	226	2.52	2	1	1	29.46	0.55	0	0	0
Anthocyanin	207	-0.76	1	0	1	13.14	0.55	0	0	0
Ribalinidine	275	2.06	4	2	0	71.7	0.55	0	0	0
Naringenin	272	1.75	5	3	1	86.99	0.55	0	0	0
Spartein	234.	3.12	2	0	0	6.48	0.55	0	0	0
Sapogenin	491	4.07	5	4	2	90.15	0.55	0	0	0
Phenol	94	1.24	1	1	0	20.23	0.55	0	0	0
Flavonones	224	1.27	2	0	1	26.3	0.55	0	0	0
Steroid	392	2.26	6	3	2	94.8	0.55	0	0	0
Epicatechin	290	1.47	6	5	1	110.4	0.55	0	0	0
Kaempferol	286	1.7	6	4	1	111.1	0.55	0	0	0
Phytate	660	-4.78	24	12	12	459	0.10	3	2	1
Flavone	222	2.55	2	0	1	30.2	0.55	0	0	0
Oxalate	88	-0.4	4	0	1	80.3	0.55	0	0	0
Catechin	290	1.47	6	5	1	110.4	0.55	0	0	0
Resveratrol	228	1.71	3	3	2	60.69	0.55	0	0	0
Tannin	729	1.89	21	14	12	371	0.17	3	2	1

**Key**: MW = molecular weight (g/mol); Log P = octanol-water partition coefficient; HBA = hydrogen bond acceptor; HBD = hydrogen bond donor; TPSA = topological polar surface area; Nro = # rotatable hydrogen bond; BAS = bioavailability score.

**Fig 6:**
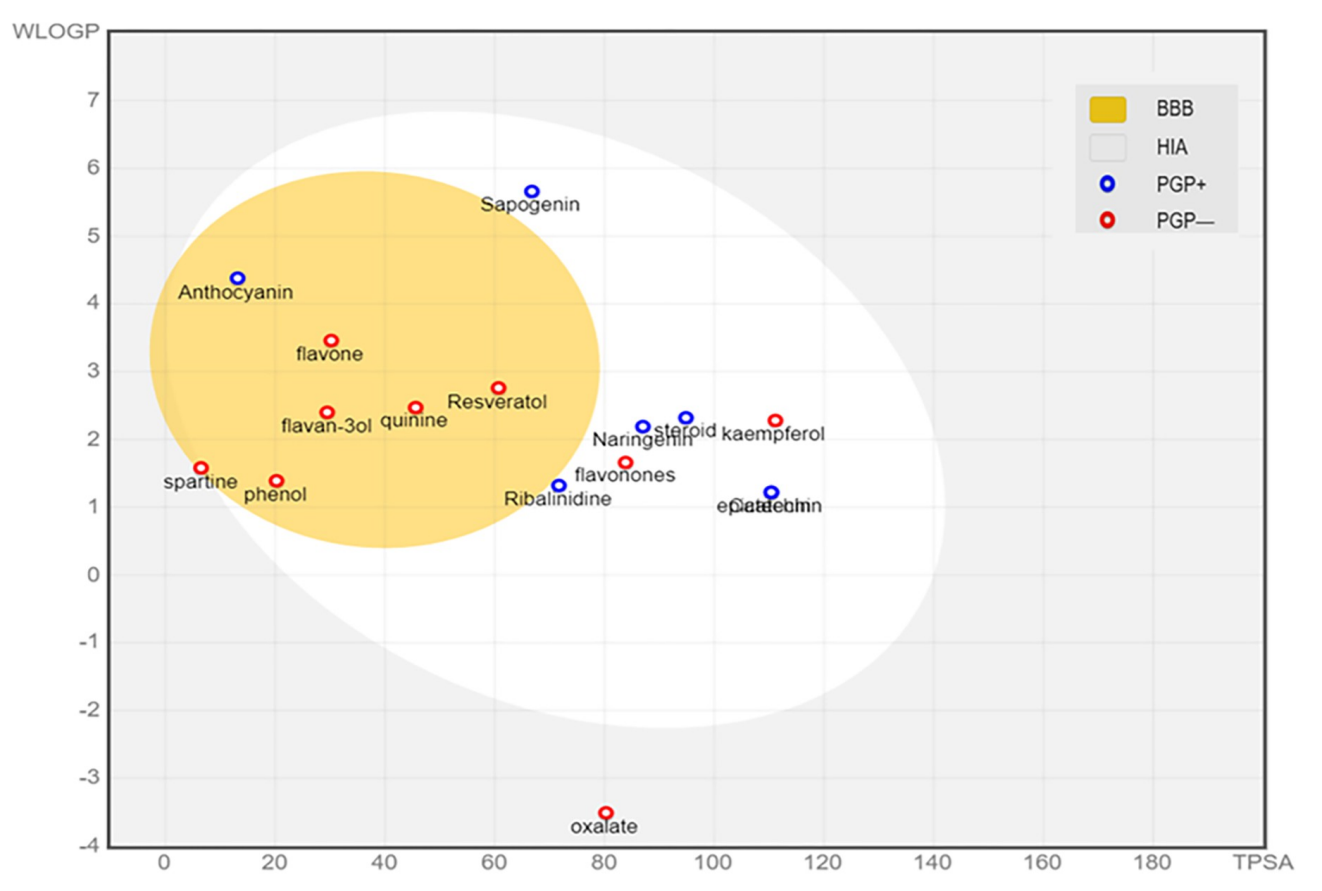
BOILED Egg model to predict GI absorption and brain permeability of the compounds identified from *Terminalia catappa nuts*. **Key:** BBB; Blood-brain barrier (compounds that can penetrate BBB found inside yellow part of the egg), HIA; Human intestinal absorption (compounds that are absorbed by the human intestine in the white part of the egg), PGP^+^, P-glycoprotein substrate (blue circle on top of PGP+ substrates) and PGP^-^; P-glycoprotein inhibitor (red circle on top of PGP^-^ inhibitors).

As shown in **Fig 6**, the BOILED egg model helped to predict the GI absorption and brain permeability of the compounds identified from *T*. *catappa nuts*. Most compounds were found to exhibit high GI absorption, with the exception of proanthocyanin, naringin, sparteine, phytate, oxalate and tannin. The blood-brain barrier (BBB) that acts as "physical" and "biochemical" (e.g., p-glycoproteins) barriers would shield the brain from the influx of xenobiotics. Bypassing the barriers would be crucial for drugs that target the neurological system [64]. Also shown in **Fig 6**, the predicted BBB permeation from WLogP and TPSA revealed anthocyanin, flavone, flavan-3-ol, quinine, resveratrol, phenol, flavonones and sparteine as brain permeants, which further suggested some promise as pharmaceutical agents especially useful in tackling neurological diseases. To ascertain if the potential drug would be orally absorbed, and to either cause minimal/no side effects, or emerge target-tissue(s) specific, should be among the fundamental pharmacokinetics properties [62]. Moreover, two central pharmacokinetics behavior considered as crucial, which are estimated at various stages of the drug discovery process, would include gastrointestinal absorption (GIA) and brain access [34]. Thus, such compounds with a high GIA upon oral administration should be absorbed with ease particularly within the intestinal tract. Rather than the oral administration, the drugs with a low GIA would be best administered through alternative routes.

The pharmacokinetics prediction output of compounds identified from *T*. *catappa* nuts is shown in **Table 7**. Naringin, sparteine, sapogenin, steroid, epicatechin, phytate, oxalate, catechin, and tannin appear unable to inhibit any of the cytochrome P450 (CP450) isoforms, whereas the others were able to inhibit at least 2 of the isoforms. Skin permeation (Log kp) value is key when evaluating drugs that might require transdermal administration, and compounds with Log kp > - 2.5 associated with low skin permeability [65]. Such identified compounds, as found herein, would be suggesting “skin permeants”, given the Log kp values < -2.5, which proffers CYP450 to possess metabolizing enzymes that are involved in drug biotransformation within the hepatocytes. By enhancing the drug-drug interactions, therefore, the inhibition of CYP450 isoforms should bring about unwarranted adverse effects and toxicity, which appear largely linked to either (drug) accumulation or lower clearance [66]. Whilst acute toxicity (LD_50_) and its classification remain vital in the discovery of doses, there would be harmful effects that emanate from potential drug candidates with lower LD_50_ (classes 1–3 with higher lethal effects) [67]. In this current work also, the predicted toxicity of compounds was identified from *T*. *catappa* nuts, as shown in **Table 8**. Most compounds that found LD_50_ > 2000 mg/kg should be considered as non-toxic and relatively harmless (classes 5 and 6). As the lowest predicted LD_50_ value suggests body weight of 150 mg/kg, a compound like ribalinidine should serve as a p-glycoprotein substrate. Moreover, the efflux protein should prevent the cellular compounds from accumulating and thereby attaining toxic levels [62]. Nonetheless, no identified compound at this current study would predict a hepatotoxic effect, which suggested *T*. *catappa* nut likely not able to harm the liver. If the opposite were to be the situation, that is, the ability to deemed to harm the liver, such would be among the major reason(s) for drug rejection, and subsequent withdrawal from the market [68]. Besides, the prediction of carcinogenicity, immunogenicity, mutagenicity, and cytotoxicity helps to ascertain the undesirable effects of a drug compound on the deoxyribonucleic acid (DNA), cells, and immune system [68]. Further, these (above-mentioned) identified compounds found in the *T*. *catappa* nuts should not be seen as either completely carcinogenic, immunogenic, mutagenic, or cytotoxic. Both slight optimization and correct dosing should help bypass the toxicity profiles of compounds with low LD_50_. More so, the synergistic activity of these phyto-constituents might mask the predicted toxic effects of the few identified compounds.

**Table 7:** Pharmacokinetics prediction output of compounds identified from raw and roasted *Terminalia catappa* nuts.

Compounds	P-gp substrate	Inhibitors of					Log kp
		CYP1A2	CYP2C19	CYP2C9	CYP2D6	CYP3A4	
Proanthocyanin	No	No	No	No	No	Yes	-8.0
Naringin	Yes	No	No	No	No	No	10.2
Quinine	No	No	No	No	Yes	No	-6.2
Flavan-3-ol	No	No	No	No	Yes	No	-5.7
Anthocyanin	Yes	Yes	No	No	Yes	No	-5.1
Ribalinidine	Yes	Yes	No	No	No	No	-6.9
Naringenin	Yes	Yes	No	No	No	Yes	-6.2
Spartein	No	No	No	No	No	No	-5.9
Sapogenin	Yes	No	No	No	No	No	-6.6
Phenol	No	Yes	No	No	No	No	-5.8
Flavonones	No	Yes	No	No	No	No	-5.4
Steroid	Yes	No	No	No	No	No	-7.3
Epicatechin	Yes	No	No	No	No	No	-7.8
Kaempferol	No	Yes	No	No	Yes	Yes	-6.7
Phytate	Yes	No	No	No	No	No	-17.6
Flavone	No	Yes	Yes	No	No	No	-5.1
Oxalate	No	No	No	No	No	No	-7.0
Catechin	Yes	No	No	No	No	No	-7.8
Resveratrol	No	Yes	No	Yes	No	Yes	-5.5
Tannin	Yes	No	No	No	No	No	-11.7

**Key**: Pgp = P-glycoprotein; CYP450 = Cytochrome P450; Log kp = skin permeation.

**Table 8:** Toxicity prediction of compounds identified from raw and roasted *Terminalia catappa* nuts.

Compounds	LD_50_ (mg/kg)	Toxicity class	Hepato- toxicity	Carcino- genicity	Immuno- genicity	Muta- genicity	Cyto- toxicity
Proanthocyanin	2500	5	-	-	+	-	-
Naringin	2300	5	-	-	+	-	-
Quinine	263	3	-	-	+	-	-
Flavan-3-ol	2500	5	-	-	-	-	-
Anthocyanin	2500	5	-	+	-	+	-
Ribalinidine	150	3	-	-	+	+	-
Naringenin	2000	4	-	-	-	-	+
Spartein	220	3	-	-	+	-	-
Sapogenin	2500	5	-	+	+	-	-
Phenol	270	3	-	-	-	-	-
Flavonones	2000	4	-	+	-	-	+
Steroid	3000	5	-	-	+	-	-
Epicatechin	10000	6	-	-	-	-	-
Kaempferol	3919	5	-	-	-	-	-
Phytate	1500	4	-	-	-	-	-
Flavone	2500	5	-	+	-	-	-
Oxalate	660	4	-	-	-	-	-
Catechin	10000	6	-	-	-	-	-
Resveratrol	1560	4	-	-	-	-	-
Tannin	2260	5	-	-	-	-	-

**Key**: LD_50_ = lethal median dose; (+) = Active; (—) = inactive.

## Conclusions

The changes in nutritional, health benefits, and pharmaceutical potential of raw and roasted *T*. *catappa* nuts from Nigeria were investigated. Findings from this current study revealed that *T*. *catappa* contains promising bio-nutrients essential for maintaining good health, which places it as a nutritional supplement with pharmaceutical potential able to ameliorate nutrient-related diseases. Not only to influence the fat-soluble vitamin, mineral content and flavor, the roasting processing method would reduce the anti-nutrients that aid absorption and palatability of the nuts. Furthermore, the findings from this current study provided evidence regards the predicted drug-likeness, pharmacokinetic properties, and good safety profile of *T*. *catappa* nuts. Nonetheless, the harvest of red almond fruits was of a geographical area as well as from a particular season could be considered a limitation at this current study. Future studies should, therefore, involve *T*. *catappa* nuts varieties harvested from different geographical locations in Nigeria, which when submitted to deeper analytically evaluation would provide additional information about the individual phytochemical components supported by *in vivo* pharmaceutical studies using animal models.
